# Molecular Insights into the Potential Insecticidal Interaction of β-Dihydroagarofuran Derivatives with the H Subunit of V-ATPase

**DOI:** 10.3390/molecules22101701

**Published:** 2017-10-11

**Authors:** Jielu Wei, Ding Li, Xin Xi, Lulu Liu, Ximei Zhao, Wenjun Wu, Jiwen Zhang

**Affiliations:** 1 College of Chemistry & Pharmacy, Northwest A&F University, Yangling 712100, Shaanxi, China; weijielu@nwafu.edu.cn (J.W.); jbolid@nwsuaf.edu.cn (D.L.); xfr92@sina.cn (X.X.); liululu4991@163.com (L.L.); ximeizhao@hotmail.com (X.Z.); 2 Key Laboratory of Botanical Pesticide R&D in Shaanxi Province, Yangling 712100, Shaanxi, China; wuwenjun@nwsuaf.edu.cn

**Keywords:** β-dihydroagarofuran, V-ATPase, subunit H of V-ATPase, isothermal titration calorimetry, ITC, fluorescence spectroscopy, molecule docking

## Abstract

Celangulin V (CV), one of dihydroagarofuran sesquiterpene polyesters isolated from Chinese bittersweet (*Celastrus angulatus* Maxim), is famous natural botanical insecticide. Decades of research suggests that is displays excellent insecticidal activity against some insects, such as *Mythimna separata* Walker. Recently, it has been validated that the H subunit of V-ATPase is one of the target proteins of the insecticidal dihydroagarofuran sesquiterpene polyesters. As a continuation of the development of new pesticides from these natural products, a series of β-dihydroagarofuran derivatives have been designed and synthesized. The compound JW-3, an insecticidal derivative of CV with a *p*-fluorobenzyl group, exhibits higher insecticidal activity than CV. In this study, the potential inhibitory effect aused by the interaction of JW-3 with the H subunit of V-ATPase c was verified by confirmatory experiments at the molecular level. Both spectroscopic techniques and isothermal titration calorimetry measurements showed the binding of JW-3 to the subunit H of V-ATPase was specific and spontaneous. In addition, the possible mechanism of action of the compound was discussed. Docking results indicated compound JW-3 could bind well in ‘the interdomain cleft’ of the V-ATPase subunit H by the hydrogen bonding and make conformation of the ligand–protein complex become more stable. All results are the further validations of the hypothesis, that the target protein of insecticidal dihydroagarofuran sesquiterpene polyesters and their β-dihydroagarofuran derivatives is the subunit H of V-ATPase. The results also provide new ideas for developing pesticides acting on V-ATPase of insects.

## 1. Introduction

**Figure 1:**
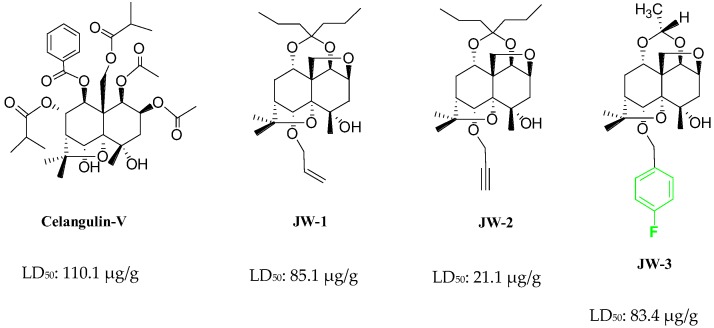
The chemical structure of Celangulin-V and some reviously reported insecticidal β-dihydroagarofuran derivatives.

Chinese bittersweet (*Celastrus angulatus* Maxim), which belongs to the family Celastraceae, has long been known for its medicinal and insecticidal properties, and lots of dihydroagarofuran sesquiterpene polyol esters extracted from this plant display excellent insecticidal activity [1,2,3,4,5,6,7]. These insecticidal compounds mainly affect the digestive system of pests, which after oral administration present a series of symptoms, such as excitement, twitching, emesis, and loss of body fluid [8]. Transmission electron microscopy (TEM) analysis show that celangulin V (CV) could induce time-dependent cytotoxicity in the midgut epithelial cells of *Mythimna separata* Walker larvae, such as visible vacuolization of cytoplasm, serious disruption of microvilli, fragmentation of RER cisternae, and rupture of plasma membrane. Subsequently, these morphological changes induce leakage of cytoplasm contents into the midgut lumen, resulting in the appearance of numerous lysosome-like vacuoles and secretion [8]. And as a continuation of the development of new pesticides from these natural products, we have previously reported the design and synthesis of a series of β-dihydroagarofuran derivatives, which gave a good number of hit compounds through screening against the larvae of *Mythimna separata* Walker [9,10]. Moreover, selected lead compounds from these hits were found to exhibit excellent LD_50_ values in the range of 21.1–85.1 μg/g (Figure 1). Specifically, compound JW-3, an insecticidal derivative of CV with a *p*-fluorobenzyl group, could be used as a fluorescent probe for measurement of the interaction of β-dihydroagarofuran derivatives with their target biomacromolecule.

The discovery of novel targets and new mechanisms of action is of vital significance to the development of pesticides as the discovery of novel targets may result in a series of new pesticides. Moreover, in the development of pesticides, natural products and synthetic naturally derived agrochemicals are useful probes in providing new targets [11,12]. Recently, Wu et al. have validated the subunit H of V-ATPase is one of the target proteins of the insecticidal dihydroagarofuran sesquiterpene polyesters by affinity chromatography, enzyme-inhibiting activity and microscale thermophoresis (MST) using celangulin or its derivatives [11,13]. Nevertheless, the current knowledge about the insecticidal mechanisms of celangulin V and its derivatives is still severely lacking. In addition, to the best of our knowledge, there are no studies that focus on elucidating the biological activity of the β-dihydroagarofuran derivatives of CV on the H subunit of V-ATPase at a molecular level.

Given the considerations above, in the present study, the potential insecticidal interaction of compound JW-3 with the H subunit of V-ATPase was investigated by spectroscopic techniques, isothermal titration calorimetry measurements and molecular modeling. The potential inhibitory effects caused by the interaction of JW-3 with the H subunit of V-ATPase was verified by confirmatory experiments. The binding mechanism as well as the relevant parameters, including the number of binding sites, binding constants, and binding forces were identified. Moreover, the interaction behaviors were further elucidated by a molecular docking simulation.

## 2. Results

### 2.1. Synchronous Fluorescence Spectra Studies and Quenching Mechanism Analysis

**Figure 2:**
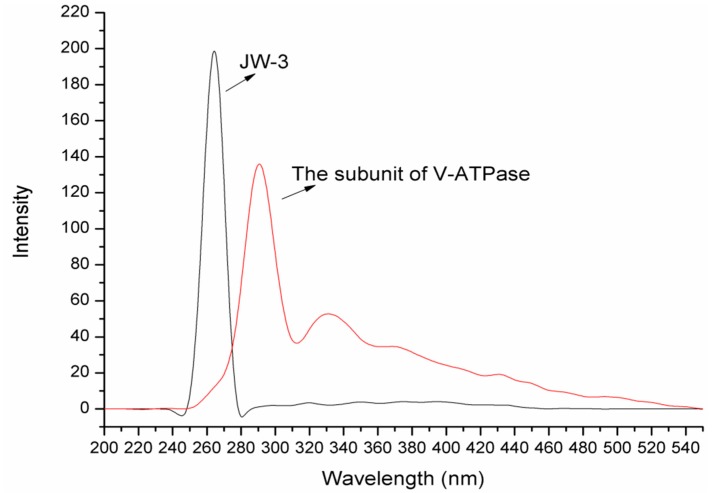
The synchronous fluorescence spectra of of both JW-3 and the H subunit of V-ATPase at a constant offset value Δλ = 15 nm.

In this study, the H subunit of V-ATPase was expressed and purified as described in the Materials and Methods section. In consideration of the fact compound JW-3 contains a fluorescent *p*-fluorobenzyl group, we first recorded the fluorescence spectra of JW-3 and the H subunit of V-ATPase by synchronous scanning at a constant offset value ∆λ = 15 nm and 60 nm. The fluorescence signal of JW-3 in the synchronous spectra was confined in a Gaussian shape with numerous narrow peaks around 260 nm at a ∆λ = 15 nm, while the fluorescence maximum of the H subunit appears above 290 nm (as shown in Figure 2). The results meant there was very low spectral overlap between the compound and protein. This observation also indicated that synchronous fluorescence spectra may provide a simple way of estimating the binding interaction between JW-3 and the H subunit of V-ATPase. It was worth mentioning here that the fluorescence signal of JW-3 was very weak by synchronous scanning at a ∆λ = 60 nm, so for the rest of this work, we chose a ∆λ = 15 nm.

**Figure 3:**
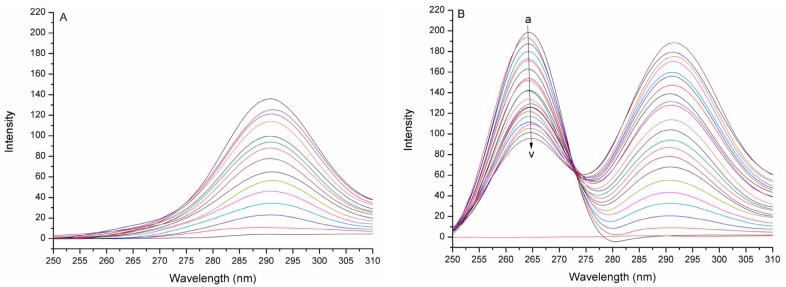
The fluorescence quenching spectra of JW-3 by the subunit H of V-ATPase at 298 K and pH 6.8. (**A**) In the absence of JW-3; (**B**) In the constant concentration of JW-3 (6.578 × 10^−5^ M). The concentration of the protein changed from a to v (10^−7^ M): 0, 0.67, 2.00, 3.32, 4.62, 5.90, 7.17, 8.42, 9.66, 10.88, 12.08, 13.27, 14.45, 15.61, 16.75, 17.89, 19.00, 20.11, 21.20, 22.29, 23.35, 24.41.

The synchronous fluorescence spectra of JW-3 following addition of different concentrations of the H subunit of V-ATPase are shown in Figure 3. To gain insights into the nature of the interactions between JW-3 and the protein, the fluorescence quenching profile of JW-3 was modeled with the following Stern-Volmer equation [14]:

(1) $$\frac{F_{0}}{F}=1+K_{sv}\left[Q\right]$$

where *F*_0_ and *F* are the steady-state fluorescence intensities in the absence and presence of a quencher, respectively; [Q] is the concentration of the quencher; *K_sv_* is the Stern-Volmer dynamic quenching rate constant. Based on the linear fit plot of *F_0_*/*F* versus [Q], the *K_sv_* values and Stern-Volmer curves of JW-3—the subunit H system could be obtained.

It is well known that fluorescence quenching is the decrease of the fluorescence quantum yield from a fluorophore induced by a variety of molecular interactions, such as excited-state reactions, energy transfer, ground-state complex formation, and collisional quenching. The quenching mechanisms are usually classified as dynamic quenching and static quenching, which can be distinguished by their different dependence on temperature and viscosity. In dynamic quenching, increasing the temperature results in faster diffusion and, hence, increased collision, thereby raising the quenching constant. In contrast, in static quenching, increasing the temperature weakens the stability of the formed complex and, hence, reduces the quenching constant. Thus, additional experiments were conducted.

**Figure 4:**
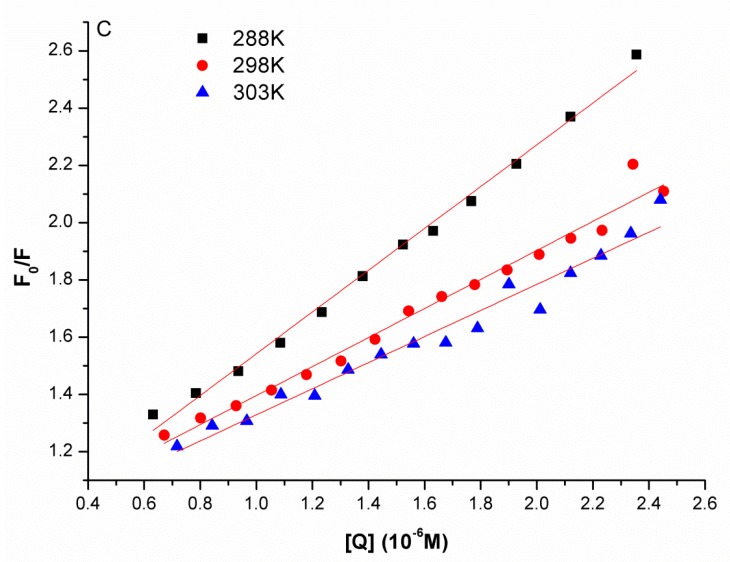
Stern-Volmer plots describing JW-3 quenching caused by the subunit H of V-ATPase at three different temperatures.

The Stern-Volmer plots at different temperatures are shown in Figure 4, and the *K_sv_* values derived from Equation (1) at the three temperatures were presented in Table 1. It was found that the *K_sv_* value decreased when the temperature rose from 288 K to 303 K, which indicated that the probable binding interaction of JW-3 with the H subunit of V-ATPase was static quenching by complex formation, rather than dynamic collision.

**Figure 5:**
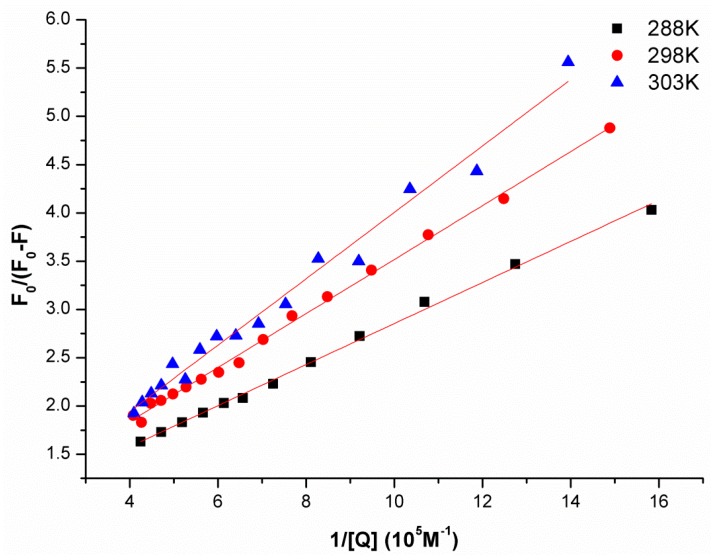
The modified Stern-Volmer plots used to obtain association constant of the subunit H of V-ATPase as reported method [15]. The temperature is as indicated in the figure.

Additionally, the quenching process was further analyzed using the following modified Stern-Volmer equation [15]:

(2) $$\frac{F_{0}}{F_{0}-F}=\frac{1}{f_{a}K_{a}}\frac{1}{Q}+\frac{1}{f_{a}}$$

where, for our study, *F*_0_ and *F* are the fluorescence intensity in the absence and presence of the quencher, respectively; *K_a_* is the effective quenching constant for the accessible fluorophores, which is analogous to the association binding constants (*K_a_*) for the quencher-acceptor system; [*Q*] is the concentration of the quencher; and *f*_a_ is the fraction of accessible fluorescence. As shown in Figure 5, the curves of *F*_0_/(*F*_0_ − *F*) versus [*Q*]^−1^ were linear when calculated according to quencher concentrations. The corresponding parameters are presented in Table 2. Moreover, the decreasing trend of *K*_a_ indicated that the binding of JW-3 to the V-ATPase subunit H was reduced as the temperature increased.

### 2.2. Characterization of the Binding Interaction between JW-3 and the Subunit H of V-ATPase by Isothermal Titration Calorimetry Measurements

**Figure 6:**
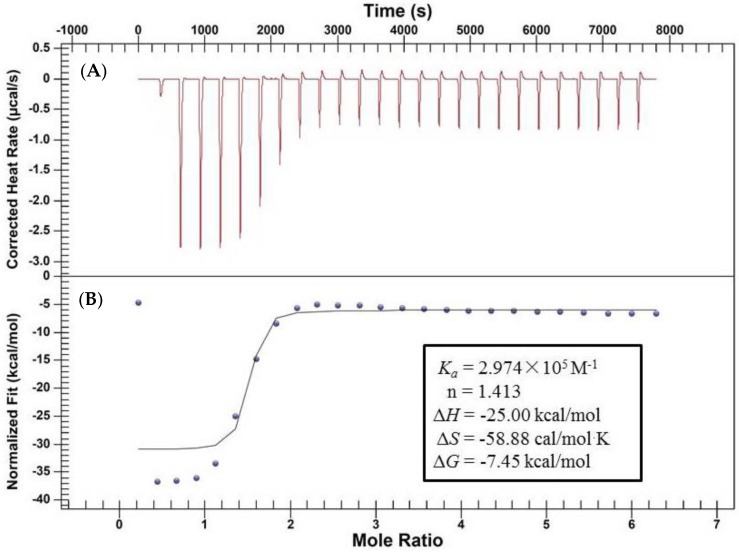
Calorimetric titration of the H subunit of V-ATPase with JW-3 at 298 K. (**A**) Heat flow as a function of time; (**B**) Reaction enthalpy of JW-3 versus injection number. The solid line corresponds to the theoretical independent model. The thermodynamic constants are presented in the pane.

The interactions between JW-3 and the H subunit of V-ATPase, as determined from spectral data, prompted us to examine the thermodynamic basis and nature of the binding forces of such interactions. To this end, we performed isothermal titration calorimetry measurements, which are widely used to study the interactions of small molecules with biomolecules. A typical titration experiment was shown in Figure 6A. After an initial exothermicity, the heats of interaction reached a constant value. The heat flows were integrated to yield the heats of reaction (Figure 6B). The heat of dilution towards the end of the system was subtracted before evaluating the data. The solid line in Figure 6B corresponded to a binding model with a 1.413:1 stoichiometry and was fitted with the change of enthalpy Δ*H* = −25 kcal/mol and entropy Δ*S* = −58.88 cal/mol·K. The equilibrium association constant (*K_a_*) between JW-3 and the H subunit of V-ATPase was also fitted and was 2.974 × 10^5^ M^−1^. This result indicated that the binding was specific. The illustration in Figure 6B contained the enthalpic and entropic components to the free energy change, Δ*G*. It was shown the spontaneous binding of JW-3 to the H subunit of V-ATPase was enthalpy-driven while the entropy was even counteracting binding.

### 2.3. Homology Modeling

The clustal analysis show that the V-ATPase subunit H from *M. separata* and yeast share 24.7% identity and 32% similarity (Figure S1 in the Supporting Information), which allows for a rather straightforward sequence alignment and guarantees the quality of homology modeling. Therefore, selecting the crystal structure of yeast V-ATPase subunit H (PDB entry code: 1ho8) as template, a plausible homology model of the target protein was generated by using SWISSMODEL server [16]. In order to reduce steric clashes and further obtain a rational modeling 3D conformation of the model structure, the staged minimizations have been further performed by using SYBYL-X2.1 as our previous studies [17,18]. The quality of homology modeling was assessed by RAMPAG [19]. The results showed that 93.9% of residues were distributed in the favoured regions, 4.7% in the allowed regions, and only 1.4% in the outlier regions, respectively (Figure S2 in the Supporting Information). These results demonstrated that the 3D structure of the *M. separata* V-ATPase subunit H was available for subsequent docking.

### 2.4. Molecular Docking

**Figure 7:**
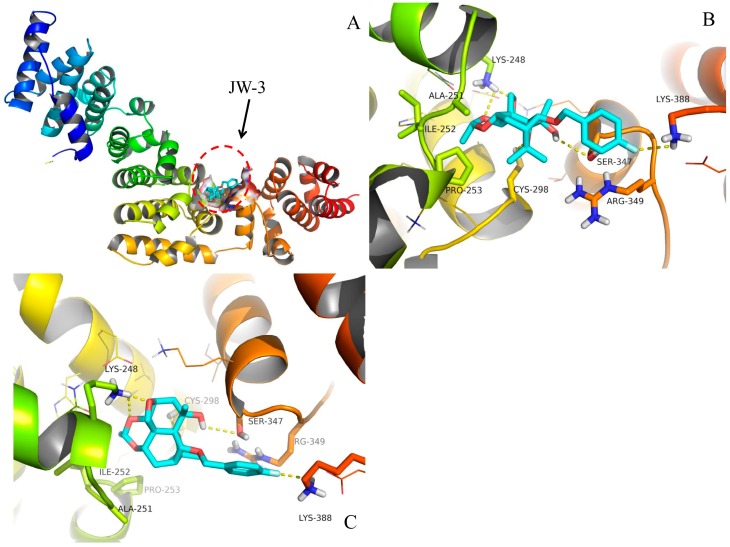
The binding model of JW-3 in the interdomain cleft of the *M. separata* V-ATPase subunit H (**A**, global view; **B**, top view; **C**, front view). Compound JW-3 (C, cyan; O, red; F, silver) were shown in sticks. The key amino acids forming the pocket were represented in sticks and lines with different color. The hydrogen bonds were denoted by yellow dash lines.

In order to understand the interaction of JW-3 with the H subunit of V-ATPase, docking simulations of the interaction between the inhibitor and the protein carried out. Docking protocol was followed with the model structure. Taking into account the high binding energy and the reasonable binding conformation, the representative configuration in the highest populated cluster with the lowest-energy was thereafter selected. As shown in Figure 7, compound JW-3 could well bind in ‘the interdomain cleft’ of the *M. separata* V-ATPase subunit H with the binding energy predicted to be −6.07 kcal/mol. The five hydrogen bonds of the hydroxyl oxygen atom with the side chain of Ser-347, the fluorine atom with the side chain of Lys-388, the three oxygen atoms on the rings with the side chain of Lys-248 and Cys-298 were reproduced (Figure 7B,C). Interestingly, the *p*-fluorobenzyl group extended into the space between the side chains of Arg-349 and Lys-388, which suggested a special electrostatic interaction. It is believed that these polar contacts were the major contributors to the stabilization of the ligand binding.

## 3. Discussion

Synchronous fluorescence spectroscopy (SFS) through synchronous scanning of both excitation and emission wavelengths is known to provide narrower and more symmetric spectra in a wider spectral range. This scan functionality results in a higher spectral selectivity for the monitoring of fluorescence sources [20,21]. By reducing spectral bleed through, SFS serves as a simple and useful method for the simultaneous determination of fluorescent components in complex mixtures. Also, SFS can be used to measure fluorescence quenching and provide information regarding the molecular environment in the vicinity of the chromophore molecules, so synchronous fluorescence spectroscopy usually plays an important role in studies on interaction between proteins and small molecules [20,21,22,23]. Traditionally, researchers focus on the fluorescence quenching of proteins induced by small ligands. However, in this study, discernible changes of the fluorescence signal were not recorded as JW-3 was titrated into solutions of the H subunit of V-ATPase. There are two possible reasons for such a phenomenon. Firstly, the emission spectrum of JW-3 and the excitation spectrum of the protein are partially overlapped (seen Figure 2). This means the emitted light of JW-3 could excite intrinsic fluorophores of the protein and the increased concentration of the compound will strengthen the fluorescence of the protein. Secondly and most importantly, there are eight tryptophan (Trp), twelve tyrosine (Tyr), and sixteen phenylalanine (Phe) residues in the protein. When it interacts with other compounds, the microenvironment change of the individual intrinsic fluorophore (Trp, Tyr or Phe) with the ligand's concentration does not have an apparent effect on the whole fluorescence of the protein. It is suggested that too many intrinsic fluorophores lead to fluorescence quenching of the protein being difficult. In fact, fluorescence quenching of the protein by the compound JW-2 was not observed, similar to that by JW-3, so the fluorescence spectra of displacement experiments for the fluorescent compound JW-3 were measured at different concentrations of the protein. The fluorescence quenching mechanism revealed the binding interaction of JW-3 with the H subunit of V-ATPase was static quenching by complex formation, rather than dynamic collision.

The verification of the interaction between JW-3 and the H subunit of V-ATPase was further done by isothermal titration calorimetry measurements and molecular docking studies. The association constants determined (*K_a_*) by ITC was 2.974 × 10^5^ M^−1^ and a value of the same order of magnitude was measured by fluorescence titration. The differences in *K_a_* values between ITC and fluorescence spectroscopy must be attributed to the different assay conditions. In addition, the ITC experiments presented the binding of the inhibitor to the subunit H of V-ATPase was driven by enthalpy, but the entropy term even counteracted the binding. It was another example of the enthalpy−entropy compensation effect on the ligand−protein interaction. The interpretation of Δ*H* and TΔ*S* in terms of molecular structure was difficult, but it is common to associate Δ*H* with van-der-Waals and electrostatic interactions [24]. However, as these were increased, the molecular structure of the ligand–protein complex rigidified and the loss in conformational freedom produced a negative TΔ*S* term which reduced the gain in enthalpy [24].

**Figure 8:**
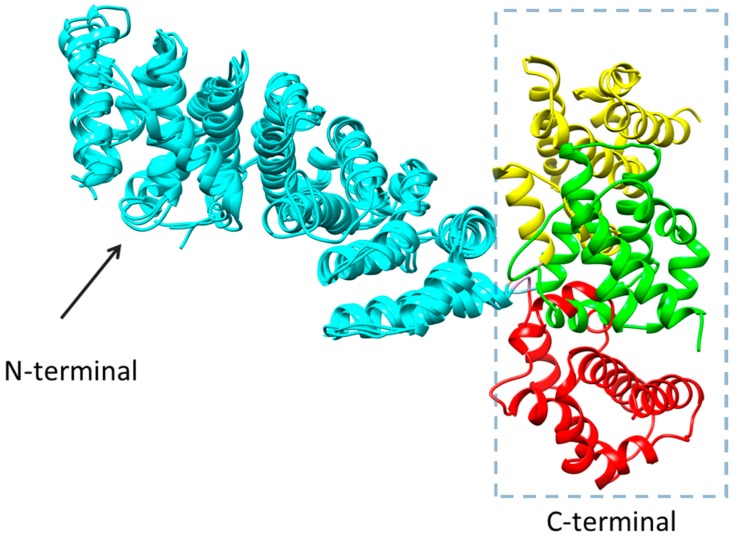
The X-ray crystallographic structures of *yeast* V-ATPase subunit H from the RCSB Protein Data Bank (PDB entry code: 1ho8, 5vox, and 5d80). All proteins are shown in cartoon model. N-terminals of all proteins are colored in cyan. C-terminals of the proteins are colored in yellow (1ho8), green (5vox), and red (5d80) respectively.

Figure 8 shows the superposition of the X-ray crystallographic structures of *yeast* V-ATPase subunit H (PDB entry code: 1ho8, 5vox, and 5d80) [25,26,27]. The three structures demonstrate the N-terminal of the protein has a relatively stable conformation, but the conformation of the C-terminal is very unstable. The C-terminal can easily transform into one of the other conformations under different conditions. Between the N- and C-terminal, a cavity could be formed by the some residues from N-terminal and the others from C-terminal, which is defined as the interdomain cleft.

Our docking results indicated compound JW-3 could well bind in ‘the interdomain cleft’ of the *M. separata* V-ATPase subunit H. The compound JW-3, like two hands, pulled both terminals of the protein and brought the loss in conformational freedom of the ligand–protein complex. In this way, the combination of the H subunit and whole V-ATPase complex may be affected, and then the function of V-ATPase complex may be destroyed. The binding model of JW-3 could well help us to understand the reduction of entropy in ITC experiment. Moreover, the polar contacts (such as hydrogen bonding, Figure 7) contributed the change of enthalpy, Δ*H*, during the binding of JW-3 to the protein. In all, both the results of isothermal titration calorimetry measurements and molecular docking were consistent with each other, and verified the binding between JW-3 and the subunit H of V-ATPase was specific.

## 4. Materials and Methods

### 4.1. Materials

*E. coli* BL21 (DE3) which can express the subunit H of V-ATPase of *M. separata* was provided by Institute of Pesticide Science, NWAFU (Yangling, China). Compound JW-3 (purity > 98% according to HPLC analysis (shimadzu corporation, Shanghai, China) was synthesized by our previously reported method [11]. Ni-NAT agarose was purchased from GE Healthcare (Beijing, China). All the other chemicals were of analytical grade, and were purchased from commercial suppliers. All solutions were prepared with ultrapure water.

### 4.2. Expression and Purification of the Subunit H of V-ATPase

The expression and purification of the H subunit of V-ATPase were determined according to our previous protocol with minor modifications [11]. The eluted proteins washed with the elution buffer (20 mmol/L Tris-HCl, 300 mmol/L NaCl, 500 mmol/L imidazole, pH 8.0) were further washed with wash buffer (20 mmol/L Tris-HCl, 300 mmol/L NaCl, pH 8.0) and concentrated using an Ultrafiltration Cup with 10 kDa Ultrafiltration Membrane Discs (EMD Millipore Corporation, Billerica, MA, USA). The final purity (>95%) of the sample was verified by SDS-PAGE electrophoresis and the concentrations of purified proteins were determined by the Bradford method. The final purified protein was stored in 20% (*v*/*v*) glycerol at −22 °C.

### 4.3. Fluorescence and Synchronous Fluorescence Measurements

All fluorescent measurements were carried out on a LS55 Fluorescence Spectrometer (PerkinElmer Inc., Waltham, MA, USA) equipped with a xenon lamp source and 1.0 cm path length quartz fluorescence cuvette. Synchronous fluorescence spectrum was obtained by simultaneously scanning the excitation and emission monochromators at a constant offset value ∆λ = λ_em_ – λ_ex_ = 15 nm and 60 nm. It was recorded over a wavelength range of 200–650 nm in the absence and presence of various concentrations of V-ATPase Subunit H in 67 mM phosphate buffer (pH 7.9, mixture of NaH_2_PO_4_·2H_2_O and Na_2_HPO_4_·12H_2_O). The excitation and emission slit widths were set at 5 nm. The scan speed and PMT voltage were 1000 nm/min and 650 V, respectively. The appropriate blank corresponding to the buffer was subtracted to correct background of fluorescence.

### 4.4. Isothermal Titration Calorimetry (ITC) Experiment

Titration of JW-3 into the V-ATPase subunit H was performed with a Nano ITC(SV) instrument (TA Instruments Ltd, Crawley, West Sussex, UK). All solutions were prepared in 20 mM Tris-Base buffer adjusted to pH 7.8. The V-ATPase Subunit H solution (8.63 μM) was placed in the 950 μL sample cell of the calorimeter, and 0.2 mM JW-3 solution with a final concentration of 0.1% DMSO was loaded into the injection syringe. The JW-3 was titrated into the sample cell at 298 K as a sequence of 25 injections of 10 μL. The time delay between injections was 300 s. The content of the sample cell was stirred at a speed of 300 rpm/min throughout the experiment to ensure comprehensive mixing. Control experiments included the titration of JW-3 solution into Tris-Base buffer.

### 4.5. Data Analysis

Raw data from the ITC instrument were obtained as plots of heat (μcal) against mole ratio and exhibited a series of peaks for each injection. The raw data were transformed using NanoAnalyze Data Analysis software (version 3.7.5., TA Instruments) to obtain a plot of observed enthalpy change per mole of injectant (ΔH kcal/mol) against the molar JW-3/protein ratio [28]. The estimated binding parameters were obtained from ITC data using the same NanoAnalyze Data Analysis software. Data fits were obtained in an independent-model way.

### 4.6. Homology Modeling

A homology model structure of the V-ATPase subunit H of *M. separata* was built using the SWISSMODEL server as described in our previous study [17,18]. Briefly, the query amino acid sequence (accession No.: AHF70968) was entered as the input parameter. The X-ray crystallographic structure of yeast V-ATPase subunit H (PDB entry code: 1ho8) was selected as template. The amino acid sequence of the query was aligned with the template protein. The homology model was built by inheriting the backbone conformation from the structural template and replacing non-identical side chains while preventing the change of as many torsion variables as possible. Subsequently, all hydrogen atoms were subsequently added to the unoccupied valence of heavy atoms of the model structure at the neutral state by using the UCSF Chimera software [29].

### 4.7. Molecular Docking

Docking studies were performed by using the AutoDock 4.2 software as our previous studies [18]. The preparations relevant to Autodock docking were done using the Autodock Tools. The ligand rigid roots were automatically set and all possible rotatable bonds and torsions were defined as active. The grid box (40 × 40 × 40) was set according to the interdomain cleft. For random simulation, the grid box was enlarged. The docking parameters consisted of setting the population size to 150, the number of generations to 270,000, and the number of evaluations to 25,000,000 while the number of docking runs was set to 20 with other default values during each docking run. Docking calculations were carried with the Lamarkian Genetic Algorithm (LGA). Pymol and UCSF Chimera was used to display the conformation and interaction [30].

## 5. Conclusions

The correlation analyses of the binding interaction between JW-3 and the H subunit of V-ATPase showed the binding of JW-3 to the subunit H of V-ATPase was specific and spontaneous. The association constant was about 2.974 × 10^5^ M^−1^. The enthalpy change induced by the electrostatic interaction drove the binding while the entropy was even counteracting binding. Our docking results indicated compound JW-3 could well bind in ‘the interdomain cleft’ of the V-ATPase subunit H and then make conformation of the ligand–protein complex become more stable. All results are the further validations of the hypothesis, that the target protein of insecticidal dihydroagarofuran sesquiterpene polyesters and their β-dihydroagarofuran derivatives is the subunit H of V-ATPase.

## Figures and Tables

**Table 1:** The Stern-Volmer quenching constant *K_sv_* of JW-3-V-ATPase subunit H system at different temperatures were deduced from Figure 4.

pH	Temperature/K	*K_SV_* (M^−1^)	R^2^
	288	7.295 × 10^5^	0.9935
6.8	298	5.070 × 10^5^	0.9799
	303	4.557 × 10^5^	0.9662

**Table 2:** Modified Stern-Volmer association constant (*Ka*) deduced from Figure 5.

pH	Temperature/K	*Ka* (M^−1^)	R^2^
	288	3.42 × 10^5^	0.9974
6.8	298	2.58 × 10^5^	0.9970
	303	1.65 × 10^5^	0.9840

## Supplementary Materials

Supplementary materials are available online.

## Abbreviations

CV	Celangulin V
V-ATPase	Vacuolar H^+^ATPase
TEM	Transmission electron microscopy
ITC	Isothermal titration calorimetry
SFS	Synchronous fluorescence spectra
