# Supplementary material for: Molecular Insights into the Potential Insecticidal Interaction of β-Dihydroagarofuran Derivatives with the H Subunit of V-ATPase
Source: Molecules. 2017 Oct 11;22(10):1701. doi: 10.3390/molecules22101701 (PMC6151423; doi:10.3390/molecules22101701)
Supplement: Supplementary file 1 [file molecules-22-01701-s001.pdf]

Molecular Insights into the Potential Insecticidal Interaction of β-Dihydroagarofuran Derivatives with the Subunit H of V-ATPase

Jielu Wei[1, †], Ding Li[1, †], Xin Xi[1], Lulu Liu[1], Ximei Zhao[1],Wenjun Wu[2], Jiwen Zhang[1,2,*]

1 College of Chemistry & Pharmacy, Northwest A&F University, Yangling, Shaanxi, 712100,

P. R. China

2 Key Laboratory of Botanical Pesticide R&D in Shaanxi Province, Yangling, Shaanxi, 712100, P. R. China

[*]Corresponding author (Tel: +86-029-87092191; Fax: +86-029-87093987; E-mail: nwzjw@nwsuaf.edu.cn)

[†] These authors contributed equally to this work.

Table of contents

Figure S1 S1

Figure S2 S2

CLUSTAL O(1.2.4) multiple sequence alignment

```
query           MANIGDGNVSQLMPPLGDEKIDMIAATSALQIRASEIRQENITWSSYLQSQMITQRDHDF
1ho8            -------------GSMGATKILM--DSTHFNEIRSIIRSRSVAWDALARSEELSEIDAST
                               :*  ** *   :: ::   * **...::*.:  :*: ::: * .

query           IVNL-------DRGQHKDLPDKNP-----ELCAEVFLN---MLTHISKDNTIQYLLVMID
1ho8            AKALESILVKKNIGDG--LSSSNNAHSGFKVNGKTLIPLIHLLSTSDNEDCKKSVQNLIA
                   *       : *:   * ..*      :: .:.::    :*:  .:::  : :  :*

query           DILSEDK---NRVKIFREGR-----------TGNAWQPFLNLLNRQDEFVQHMT--ARII
1ho8            ELLSSDKYGDDTVKFFQEDPKQLEQLFDVSLKGDFQTVLISGFNVVSLLVQNGLHNVKLV
                ::**.**   : **:*:*.            .*:    ::. :*  .  :**:    .:::

query           AKLACWHTTVMERSDLLFYLSWLKDQLKLNNNEYVQSVARCLQM---MLRVDEYRFA---
1ho8            EK-------LLKNNNLINI---------LQNIEQMDTCYVCIRLLQELAVIPEYRDVIWL
                 *       :::..:*:           *:* * :::   *:::   :  : *** .

query           -----------FLTV----DGISTLLAILASRVNFQVQYQLTFCLWVLTFNPLLAEKMNK
1ho8            HEKKFMPTLFKILQRATDSQLATRIVATNSNHLGIQLQYHSLLLIWLLTFNPVFANELVQ
                           :*      :  : ::*  :.::.:*:**:  : :*:*****::*::: :

query           FNAIPI--LADILSDSVKEKVTRIVLAVFRNLIEKPENQQVSKEHCIAMVQCKVLKQLSI
1ho8            KYLSDFLDLLKLVKITIKEKVSRLCISIILQCCSTRVKQHKKVI-KQLLLLGNALPTVQS
                     :  * .::. ::****:*: :::: :  ..  :*: .      ::  :.*  :.

query           LEQKRSDDEDIMNDVDFLNERLQTSVQDLSSFDQYATEVKSGRLEWSPVHKSAKFWRENA
```

```
1ho8        LSERKYSDEELRQDISNLKEILENEYQELTSFDEYVAELDSKLLCWSPPHVDNGFWSDNI
            *.::: .**:: :*:. *:* *:.. *:*:***:*.:*:.*  * *** * .   ** :*

query       IRLNERGQELLRTLVHLLEK---------SKDPVVLAVACYDVGEYVRHYPRGKHIIEQL
1ho8        DEFKKDNYKIFRQLIELLQAKVRNGDVNAKQEKIIIQVALNDITHVVELLPESIDVLDKT
             .::: . :::* *:.**:          .:: ::: **  *: . *.  *.. .::::

query       GGKQRVMHLLSHEDPNVRYEALLAVQKLMVHNWEYLGKQLEKEQIDKQAGTVVGAKA
1ho8        GGKADIMELLNHSDSRVKYEALKATQAIIGYTFK-----------------------
            ***  :*.**.*.* .*:**** *.* :: :.::
```

Figure S1. Multiple sequence alignment of the V-ATPase subunit H. from *M. separate* (query) and *yeast* (PDB:1ho8)

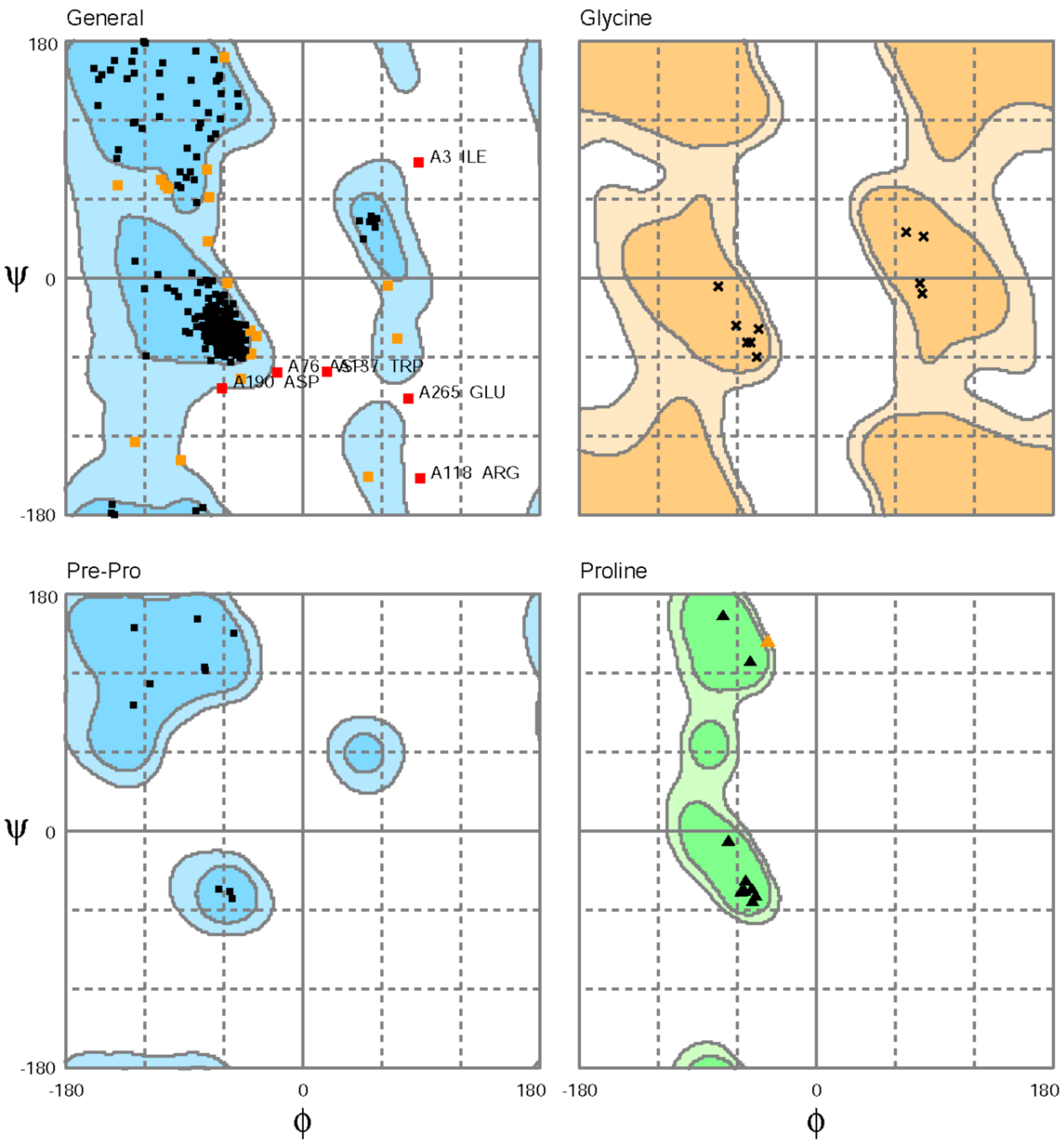

General Favoured		General Allowed	
Glycine Favoured		Glycine Allowed	
Pre-Pro Favoured		Pre-Pro Allowed	
Proline Favoured		Proline Allowed	

Number of residues in favoured region (~98.0% expected) : 403 (93.9%)
Number of residues in allowed region (~2.0% expected) : 20 (4.7%)
Number of residues in outlier region : 6 (1.4%)

Figure S2. Assessment of the Ramachandran plot of the model structure of the *M. separata* V-ATPase subunit H.
